# Performance evaluation of Active Melioidosis Detect-Lateral Flow Assay (AMD-LFA) for diagnosis of melioidosis in endemic settings with limited resources

**DOI:** 10.1371/journal.pone.0194595

**Published:** 2018-03-26

**Authors:** Tushar Shaw, Chaitanya Tellapragada, Vandana KE, David P. AuCoin, Chiranjay Mukhopadhyay

**Affiliations:** 1 Dept. of Microbiology, Kasturba Medical College, Manipal University, Manipal, India; 2 Manipal Center for Virus Research, Manipal University, Manipal, India; 3 Dept. of Molecular Microbiology and Immunology, University of Nevada, Reno, Nevada, United States of America; Tulane University School of Medicine, UNITED STATES

## Abstract

Melioidosis is a fatal infection caused by the soil saprophyte Burkholderia pseudomallei. Early diagnosis and befitting medical management can significantly influence the clinical outcomes among patients with melioidosis. Witnessing an annual increment in the number of melioidosis cases, over the past few years, mainly from the developing tropical nations, the present study was undertaken to evaluate the diagnostic utility of Active Melioidosis Detect^TM^LateralFlow Assay (AMD-LFA), in comparison with enrichment culture and PCR. A total of 206clinical specimens obtained from 175 patients with clinical suspicion of melioidosis were considered for the evaluation. Positivity for *B*.*pseudomallei* using enrichment culture, PCR and AMD-LFA were observed among 63 (30.5%), 55 (26.6%) and 63 (30.5%) specimens respectively. The AMD-LFA failed to detect melioidosis from 9 culture-confirmed cases (6 whole blood specimens, 2 pus samples, and one synovial fluid). Further the test gave faint bands from 9 urine samples which were negative by culture and PCR. AMD-LFA demonstrated a sensitivity, specificity, of 85.71%(CI:74.61% to 93.25%) and 93.62% (CI:88.23% to 97.04%), with positive predictive value of 85.71% (CI: 75.98% to 91.92%) and negative predictive value of 93.62% (CI:88.89% to 96.42%). The test needs further evaluation in view of the faint bands from negative urine samples, for incorporating the test as a point of care assay.In view of its rapidity and ease of testing AMD-LFA might be useful in early diagnosis of melioidosis at resource constraint settings.

## Introduction

*Burkholderia pseudomallei* is a soil-dwelling bacterium causing melioidosis, a disease with protean clinical manifestations. The disease is endemic in northern Australia and Northeast Thailand causing significant morbidity and mortality [1, 2]. Over the past few years, there has been an increase in the number of cases reported from the Indian sub-continent, mainly from the South Western coastal part of the country [3, 4, 5]. While the true incidence of the disease still remains unknown in India, a recent mathematical modeling has predicted the Indian sub-continent to have the highest burden of the disease [6]. Diabetes mellitus is well acknowledged as one of the important risk factors associated with a severe form of the disease [3, 4]. With a high incidence of diabetes and agriculture as one of the primary occupation practiced in the rural settings, it can be estimated that India has a high number of cases which goes misdiagnosed.

Despite mounting evidence that India is endemic for melioidosis, under/ misdiagnosis of this fatal disease is common across the country. Definitive diagnosis of melioidosis is based on culture, which takes 5 to 7 days, leading to a delay in specific medical intervention. Various serological tests such as the Indirect Hemagglutination Assay (IHA) have been widely used but the sensitivity and specificity of the test are not high enough for routine use [7]. Immunofluorescence microscopy can detect B.pseudomallei from clinical samples in<2 hours [8]. However, it requires both suitable samples, trained personnel, and specialized microscopy facilities.Hence, a cost-effective and simple diagnostic test is a requisite. The recent development of a prototype lateral flow assay for antigen detection in melioidosis gained interest as a rapid point of care test for the disease [9]. Previously, we observed and reported the diagnostic supremacy of enrichment culture and/or PCR over conventional culture for detection of B.pseudomallei from clinical specimens other than blood [5]. Lack of resources and expertise in major primary health care settings could be one of the major causes for misdiagnosis of the disease. Further, the delayed referral of the patients to the tertiary care settings lead to fatal outcomes including sepsis and organ failure. Under both the conditions, a point of care test is a requisite for early diagnosis.In our quest to further improve the case detection rates of melioidosis in resource-constrained settings, we undertook the present study to evaluate the performance of active Melioidosis detect- lateral flow assay (AMD-LFA, InBios International Inc. USA), a recently developed point-of-care diagnostic test.

## Materials and methods

Study site and specimens: The present study was undertaken at a 2030 bedded university-teaching hospital in South India. Clinical specimens including pus, respiratory secretions, sterile body fluids and whole blood samples obtained from patients suspected clinically with melioidosis during a period of two years (July 2015- June 2017) were included in the study. Clinical suspicion of melioidosis in these patients was done based on symptoms such as community-acquired pneumonia and/or septicemia, chronic illness mimicking tuberculosis or resolving infections of the skin, soft tissue, bone or joints. The study was approved by the Institutional Ethical Committee, Kasturba Hospital Manipal. Written informed consent was obtained from all subjects prior to collection of samples.

Specimen processing: All clinical specimens were subjected to routine microbiological culture techniques for isolation of bacterial pathogens. In addition, enrichment culture and conventional PCR targeting the TTSS1 region of B.pseudomallei were also performed on all the samples using previously described protocols [10].

Performance of AMD-LFA: The AMD-LFA is a lateral flow-based immunoassay that can detect capsular polysaccharide of B.pseudomallei from various sample types [9]. For use with whole blood samples, 35 μl of the samples were directly added to the test strip and then transferred to a well loaded with chase buffer, for samples like sputum, pus etc. 20 μl of sample was added to 100 μl of lysis buffer and mixed well by vortexing, 20 μl of the preparation was then added to test strip and transferred to a well-containing chase buffer. Urine samples were analyzed by centrifugation at 4000 rpm for 10 minutes, after which 20ul of the pellet was then added to the test strip. A valid positive test was indicated with the presence of a red line in both the test and control regions.

Data analysis and Statistics: Data were analyzed using SPSS version 16 (IBM, Bangalore, South Asia). Analysis established the performance of lateral flow assay in comparison to the enrichment culture. The sensitivity, specificity, positive predictive values, negative predictive values, and a measure of agreement (κ) were determined at 95% confidence interval (CI). We also used Receiver operating characteristic (ROC) curve to compare and determine the sensitivities and specificities of PCR and AMD-LFA considering enrichment culture as the reference diagnostic method.

### Results

We obtained a total of 206 clinical specimens from 175 patients with a mean age of 46.6 ± 15.8 years. Prevalence of culture-confirmed cases of melioidosis was 30.3% in our study (53/175).Two specimens from different anatomical sites were collected from 31 patients (n = 62) and a single specimen from 144 patients. Among the 31 patients with more than one clinical specimen collected for testing, 10 patients had culture-confirmed melioidosis. Majority of the specimens were pus (n = 72) followed by urine (n = 50). Details regarding the overall study specimens are shown in Table 1. Of the 206 specimens included in the study, AMD-LFA was tested within 48 hours of collection for 202 (98%) specimens, while 4 of 9 whole blood samples were stored at -70oC and tested after 60 days. Among the 206 specimens included for analysis, positivity for B.pseudomallei using enrichment culture, PCR and AMD-LFA were observed among 63 (30.5%), 55 (26.6%) and 63 (30.5%) samples respectively. Specimen negative for B.pseudomallei by any one of the methods tested, had grown other organisms like *Pseudomonas aeruginosa* (n = 17), *Staphylococcus aureus*(n = 16),*Escherichia coli* (n = 16) and*Burkholderia*cepacia (n = 5). Distribution of the study specimens based on positivity for B.pseudomallei, using all the three techniques is shown in Table 1. AMD-LFA failed to detect B. pseudomallei from 9 specimens including 6 whole blood specimen, 2 pus samples, and 1 synovial fluid. PCR failed to detect B.pseudomallei from 8 whole blood samples.

**Table 1:** Distribution of study specimens tested using enrichment culture, AMD-LFA and PCR.

Sample (N)	Culture Positive n(%)	LFA Positive n(%)	PCR Positive n(%)
Pus (72)	29(40.2)	27(37.5)	29(40.2)
Respiratory Secretion (45)	5(11.1)	5(11.1)	5(11.1)
Body Fluid (13) Peritoneal Fluid (6) Synovial Fluid (6) Pleural Fluid(3)	1(25) 1(25) 1(25)	2(33.3) 1(26.6) 2(33.3)	0 0 0
Urine (50)	15(30)	24(48)	15(30)
Tissue(5)	0	0	0
Blood Culture by BacT/ALERT(12)	3(25)	3*(25)	3*(25)
Whole Blood (9)	8(88.8)^#^	2 (22.2)	0
Total (206)	63	63	55

* Blood culture supernatant fluid after signaling positive was used for testing

^#^These 8 positive specimen depict blood culture positivity by BacT/ALERT

**Fig 1:**
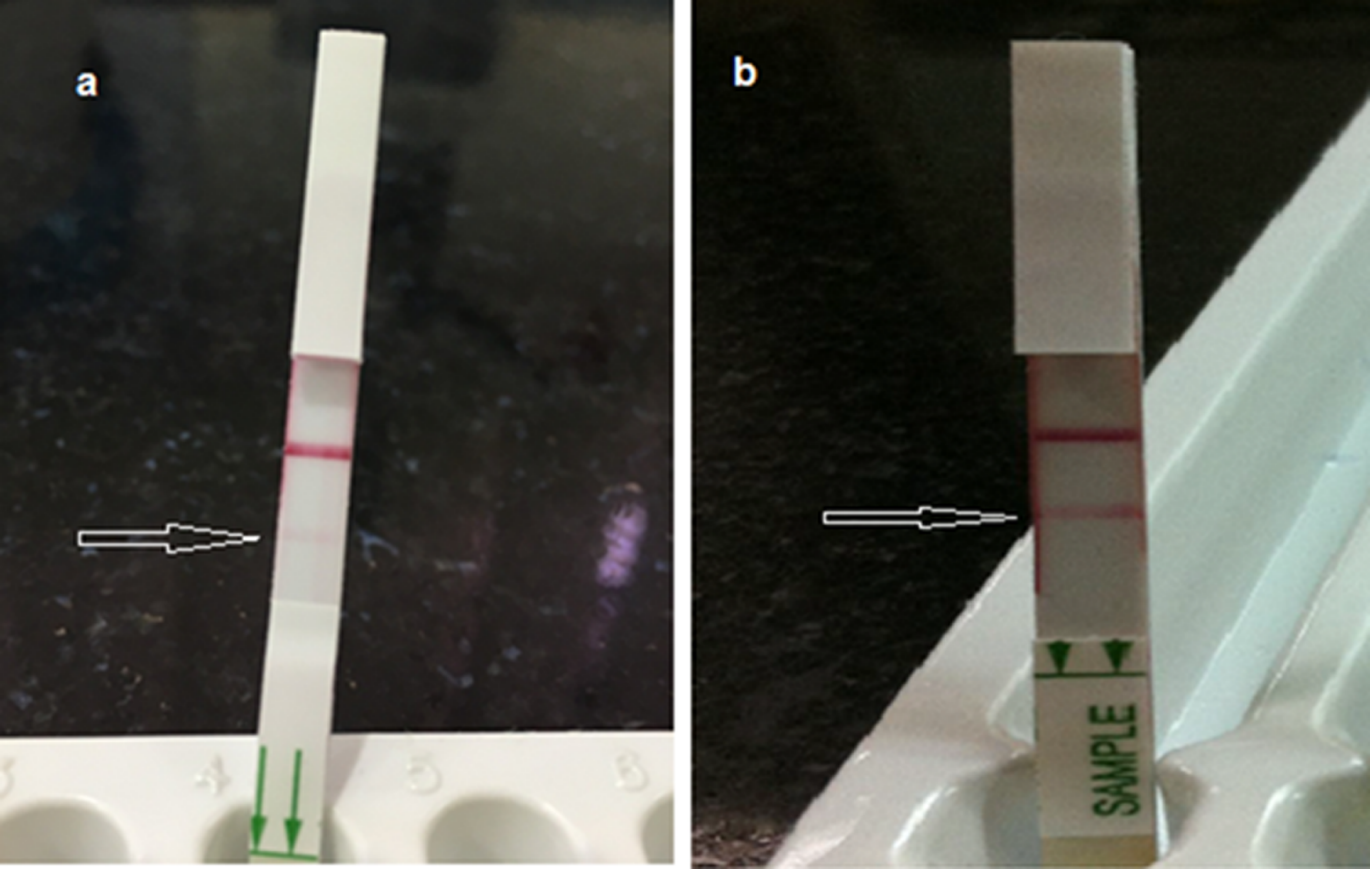
AMD-LFA test showing a) faint bands and b) strong bands.

**Fig 2:**
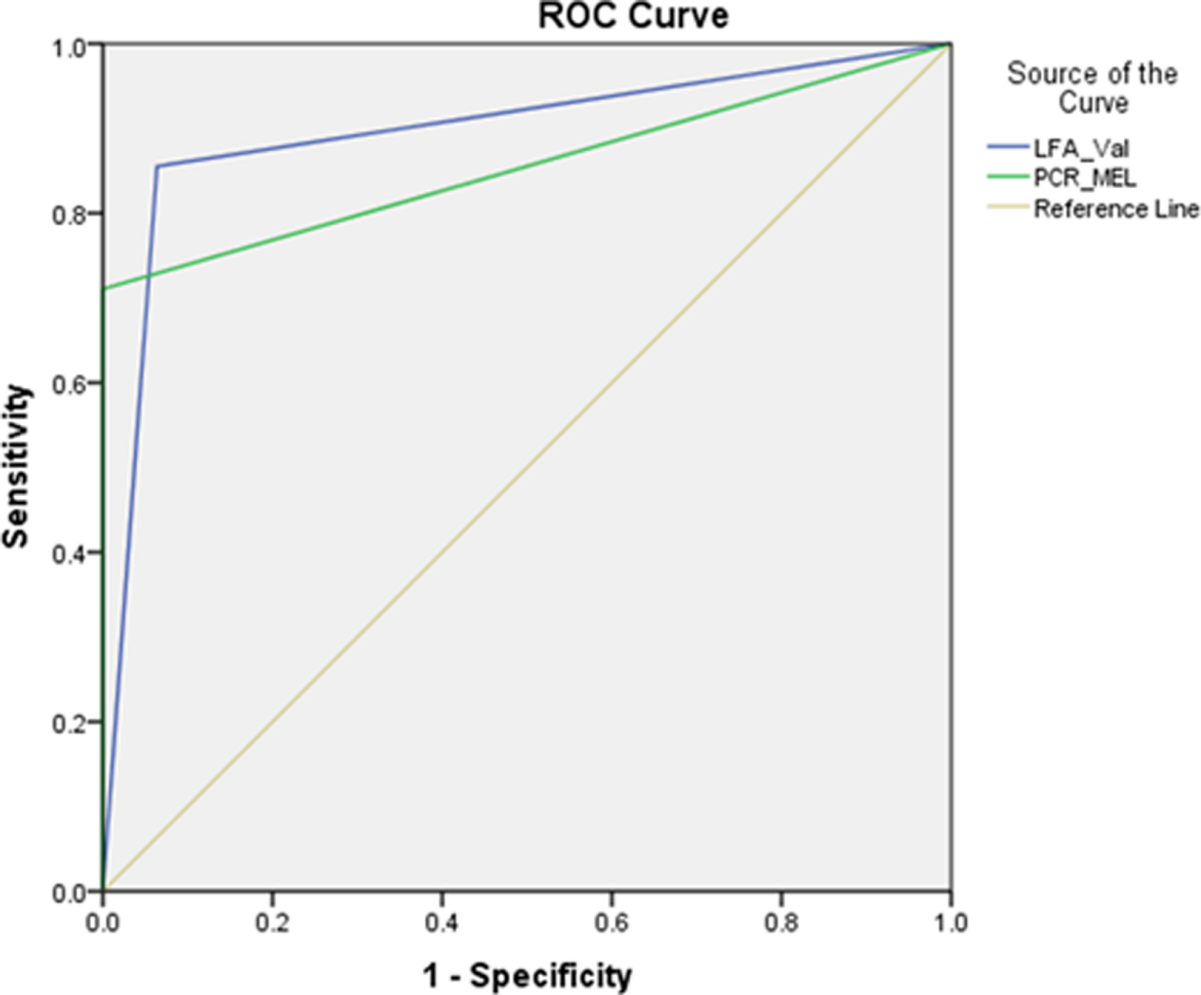
Receiver operating characteristic curve for AMD-LFA and PCR in comparison with enrichment culture.

There were 9 urine samples which showed faint band for AMD-LFA but remained negative for both culture and PCR(Fig 1).Sensitivity and specificity of AMD-LFA in comparison with enrichment culture was 85.71%(CI: 74.61% to 93.25%) and 93.62% (CI: 88.23% to 97.04%) respectively (Table 2). AMD-LFA demonstrated slightly a higher measure of agreement with PCR (κ = 0.846) than with enrichment culture (κ = 0.783). Using ROC curve, we observed similar area under the curve (AUC) values for AMD-LFA (0.896; 95% CI = 0.844–0.948) and PCR (0.855; 95% CI = 0.792–0.919) in comparison with the enrichment culture (Fig 2).

**Table 2:** Diagnostic accuracy of LFA compared with enrichment culture and PCR.

	Compared to Enrichment Culture	Compared to PCR
Sensitivity	85.71% (CI: 74.61% to 93.25%)	96.36% (CI: 87.47% to 99.56%)
Specificity	93.62% (CI: 88.23% to 97.04%)	93.24% (CI: 87.93% to 96.71%)
Positive Predictive value	85.71% (75.98% to 91.92%)	84.13% (CI: 74.40% to 90.62%)
Negative Predictive Value	93.62% (88.89% to 96.42%)	98.57% (CI: 94.65% to 99.63%)
Kappa’s coefficient	0.783; p< 0.001	0.846; p<0.001

## Discussion

In the absence of prompt diagnosis and appropriate treatment, melioidosis has high mortality. The current antibiotics of choice, ceftazidime or meropenem are expensive; they are not used empirically for community-acquired pneumonia or septicemia in developing tropical nations. Diagnosis of melioidosis mainly relies on microbiological culture and accurate identification of B. pseudomallei from clinical specimens. Under/misdiagnosis of melioidosis can be common in settings where conventional microbiological culture is used as the diagnostic test of choice. Using enrichment culture and/or PCR as adjunct diagnostic tests to the conventional culture techniques, we demonstrated an increment in the case detection rates of melioidosis in our settings previously [5]. The absence of infrastructural and human resources restricts the routine use of PCR-based diagnosis in developing countries.

The development of AMD-LFA has provided an opportunity to assess a new rapid POC diagnostic test for melioidosis. In the present study, AMD-LFA showed satisfactory results for the detection of B.pseudomallei from clinical specimens other than blood. There were 9 culture-confirmed cases which were negative by AMD-LFA including 6 whole blood specimen, 2 pus, and one synovial fluid., In case of whole blood specimens, the LFA did not perform well as compared with the automated blood culture. However, the LFA demonstrated a slightly better efficacy than PCR for the diagnosis of melioidosis from whole blood specimens. Previous reports suggest that false negative detection of B. pseudomallei from blood may be attributed due to a lower concentration of the organism in blood [11].The low volume used for testing in case of AMD-LFA and PCR could be additionally responsible for the failure of the tests to detect low levels of B.pseudomallei from clinical specimens. Except for the 9 urine specimens which showed faint bands (false positive) using AMD-LFA, we did not encounter a case where AMD-LFA was positive, but microbiological culture for B.pseudomallei was negative. The LFA has shown better results with specimens such as pus and other body fluids. There were 9 urine samples which showed faint band on AMD-LFA, but gave negative results, when tested using both culture and PCR. It is possible that the very faint positive bands observed in the 9 urine samples that were negative by culture and PCR are indeed indicative of a weak interaction by the test strip. Whether or not this corresponds to a very low load of CPS in the urine sample, indicative of a low infection threshold needs further evaluation. These nine patients were admitted to the hospital with clinical presentations suggestive of acute febrile illness. Laboratory parameters such as total leukocyte count, Differential count, and procalcitonin levels were suggestive of bacterial infection. Blood and urine cultures of these nine patients were negative for any bacterial pathogens. Serological tests for other common infectious etiology such as malaria, typhoid, brucellosis, and leptospirosis were also negative among these patients. Infectious etiology among these nine patients could not be ascertained using the currently employed diagnostic algorithms and modalities in our settings. Further followup samples were not available from these patients as all of them left the hospital before any final decisions could be made. In view of the higher burden and ease of testing clinical specimens from localized infection, the assay can be incorporated in diagnosing the disease mainly in resource-constrained settings such as the primary health-care centers.

The main limitation of this study includes testing of the limited number of fresh and archived whole blood specimens, but no serum samples. As proposed earlier [12] the use of more blood samples would have helped us determine the effectiveness of the assay in severe sepsis. The test also gave positive results from enriched blood cultures suggesting an effective avenue for rapid diagnosis of suspected melioidosis when a blood culture signals positive. This will further prevent any delay in diagnosis for critical septicemic patients, [13].

Put together, the AMD-LFA showed reasonably good sensitivity for the diagnosis of melioidosis depending on the type and combination of samples. However, considering the faint bands on the test strips, as observed in our study, we foresee a need for further evaluation of the assay. This is needed to effectively delineate false positive result from a weak interaction by the test strip due to low capsular polysaccharide load in the clinical specimen. If only this could be achieved, the test can be helpful for presumptive diagnosis of the disease and initiation of early and appropriate therapy. In view of its rapidity and ease of use, the assay can be used for screening melioidosis among patients with suggestive clinical symptoms especially in endemic areas, where standard laboratory facilities are not available.
